# 奥妥珠单抗联合化疗致急性血小板减少的初诊滤泡性淋巴瘤患者临床特征分析

**DOI:** 10.3760/cma.j.cn121090-20260225-00108

**Published:** 2026-08

**Authors:** 竟心 周, 玲 高, 奕 夏, 姝超 秦, 翰宁 唐, 祎琳 孔, 磊 范, 建勇 李, 华渊 朱

**Affiliations:** 1 江苏省人民医院宿迁医院，宿迁市第一人民医院血液科，宿迁 223800 Department of Hematology, Suqian Hospital of Jiangsu Province People's Hospital, The First People's Hospital of Suqian City, Suqian 223800, China; 2 南京医科大学第一附属医院（江苏省人民医院）血液科、淋巴瘤中心，南京 210029 Department of Hematology, Lymphoma Center, Nanjing Medical University Affiliated First Hospital, Jiangsu Provincial People's Hospital, Nanjing 210029, China

**Keywords:** 淋巴瘤，滤泡性, 奥妥珠单抗, 血小板减少, Lymphoma, follicular, Obinutuzumab, Thrombocytopenia

## Abstract

**目的:**

评估奥妥珠单抗一线免疫化疗在滤泡性淋巴瘤（FL）患者中的疗效及安全性并探讨奥妥珠单抗诱导的急性血小板减少（OIAT）的临床特征及其潜在危险因素。

**方法:**

回顾性纳入2021年1月至2024年12月江苏省人民医院收治的167例初诊FL患者，接受G-CHOP方案（奥妥珠单抗+环磷酰胺+多柔比星+长春新碱+泼尼松）或GB方案（奥妥珠单抗+苯达莫司汀）治疗。采用二元Logistic回归分析发生OIAT的影响因素。收集患者基线资料及治疗后血小板计数（PLT），记录发生OIAT患者的PLT最低值及恢复情况。

**结果:**

纳入的167例患者中，65例（38.9％）发生OIAT，其中18例（27.7％）为3～4级血小板减少。OIAT中位发生时间为首次用药后1.0（1.0，2.0）d，中位恢复时间为8.0（6.0，16.0）d。与未发生OIAT组（102/167，61.1％）相比，OIAT组基线中性粒细胞百分比［中位数：3.4（2.5，4.9）％对4.3（3.1，6.6）％，*P*＝0.005］、D-二聚体水平［中位数：0.2（0.1，0.7）mg/L对0.6（0.3，1.3）mg/L，*P*＝0.001）］、CD4⁺T细胞计数［（32.4 ± 17.6）个/µl对（37.4 ± 11.8）个/µl，*P*＝0.043］、IgA水平［中位数：1.2（1.0，2.1）g/L对1.6（1.2，2.2）g/L，*P*＝0.045］更低，而IL-10水平［中位数：8.0（3.7，32.0）ng/L对4.6（2.4，7.9）ng/L，*P*＝0.008］、β_2_微球蛋白水平［中位数：2.7（1.8，3.9）mg/L对2.2（1.7，3.0）mg/L，*P*＝0.024］更高。多因素分析显示，结外受累（*OR*＝3.395，95％ *CI*：1.229～9.378，*P*＝0.018）、FL国际预后指数-2评分>3分（*OR*＝3.622，95％ *CI*：1.192～10.973，*P*＝0.023）及基线PLT<150×10⁹/L（*OR*＝3.216，95％ *CI*：1.122～9.215，*P*＝0.030）为发生OIAT的独立危险因素。1～2级与3～4级OIAT患者中升血小板治疗组较未治疗组PLT恢复时间差异无统计学意义［1～2级：中位数：7.1（5.0，12.5）d对7.4（5.2，13.0）d，*P*＝0.338；3～4级：（21.0 ± 14.4）d对（15.5 ± 9.9）d，*P*＝0.420］；但3～4级OIAT患者较1～2级患者PLT恢复时间显著延长［中位数：16.0（8.0，18.5）d对7.0（5.0，12.8）d，*P*＝0.010］，且更易合并非血液学不良事件［发生率：72.2％（13/18）对40.4％（19/47），*P*<0.001］。

**结论:**

OIAT常见于首次用药后，基线肿瘤负荷高、免疫状态低的患者发生风险显著增加。1～2级血小板减少可观察随访，3～4级需密切监测并积极干预。识别高危患者并实施分层管理有助于增加治疗安全性。

## Abstract (en)

**Objective:**

To evaluate the efficacy and safety of first‑line immunochemotherapy with obinutuzumab in patients with follicular lymphoma（FL）, with a particular focus on the clinical characteristics and potential risk factors of obinutuzumab‑induced acute thrombocytopenia（OIAT）.

**Methods:**

The present retrospective study included 167 newly diagnosed FL patients receiving G-CHOP or GB regimens at Jiangsu Provincial People's Hospital between January 2021 and December 2024. The patients' baseline clinical characteristics and serial platelet（PLT）count were collected. In patients with OIAT, the nadir PLT count and recovery time were recorded. Logistic regression analysis was performed to identify the risk factors for OIAT.

**Results:**

Among the 167 patients, 65（38.9％）developed OIAT, including 18（27.7％）patients with grade 3–4 thrombocytopenia. The median time to OIAT onset was 1.0（1.0, 2.0）days after the first administration, and the median recovery time was 8.0（6.0, 16.0）days. Compared to patients without OIAT（102/167, 61.1％）, those with OIAT had lower baseline neutrophil percentage［median: 3.4（2.5, 4.9）％ *vs* 4.3（3.1, 6.6）％, *P*＝0.005］, lower D‑dimer levels［0.2（0.1, 0.7）mg/L *vs* 0.6（0.3, 1.3）mg/L, *P*＝0.001］, higher IL‑10 levels［8.0（3.7, 32.0）ng/L *vs* 4.6（2.4, 7.9）ng/L, *P*＝0.008］, higher β_2_‑microglobulin levels［2.7（1.8, 3.9）mg/L *vs* 2.2（1.7, 3.0）mg/L, *P*＝0.024］, lower CD4⁺ T cell counts［（32.4 ± 17.6）cells/µl, *vs*（37.4 ± 11.8）cells/µl, *P*＝0.043］, and lower IgA levels［1.2（1.0, 2.1）g/L *vs* 1.6（1.2, 2.2）g/L, *P*＝0.045］. Multivariate analysis showed that extranodal involvement（*OR*＝3.395, 95％ *CI*: 1.229–9.378, *P*＝0.018）, FLIPI‑2 score >3（*OR*＝3.622, 95％ *CI*: 1.192–10.973, *P*＝0.023）, and baseline PLT count of <150×10⁹/L（*OR*＝3.216, 95％ *CI*: 1.122–9.215, *P*＝0.030）were the independent risk factors for OIAT. In patients with grade 1–2 and 3–4 OIAT, the use of thrombopoietic therapy had no significant effect on prolonged PLT recovery time［grade 1–2: median: 7.1（5.0, 12.5）days *vs* 7.4（5.2, 13.0）days, *P*＝0.338; grades 3–4:（21.0 ± 14.4）days *vs*（15.5 ± 9.9）days, *P*＝0.420］. However, patients with grade 3–4 OIAT had significantly prolonged PLT recovery time［median: 16.0（8.0, 18.5）days *vs* 7.0（5.0, 12.8）days, *P*＝0.010］and were more likely to experience non‑hematologic adverse events［incidence rate: 72.2％（13/18）*vs* 40.4％（19/47）, *P*<0.001］.

**Conclusion:**

OIAT commonly occurs after the initial drug administration, with the risk significantly increased in patients with high baseline tumor burden and impaired immune status. Grade 1–2 thrombocytopenia may be observed during follow-up, whereas Grade 3–4 cases require close monitoring and active intervention. Identifying high-risk patients and implementing stratified management strategies can help optimize treatment safety.

滤泡性淋巴瘤（FL）是惰性非霍奇金淋巴瘤中最常见的一类，占20％～30％[1]–[3]。临床以利妥昔单抗为基础的免疫化疗方案改善了初诊FL患者的生存结局[4]–[5]。奥妥珠单抗作为一种糖基化改造的Ⅱ型抗CD20单抗，具有更强的抗体依赖性细胞毒性，Ⅲ期GALLIUM研究结果显示其无进展生存优于利妥昔单抗[6]–[7]。中国患者亚组也证实奥妥珠单抗联合化疗的3年无进展生存率较利妥昔单抗联合化疗更高（81.8％对70.2％）[8]，美国国家综合癌症网络临床实践指南及中国指南均推荐初治FL患者接受奥妥珠单抗联合化疗[8]–[9]。

随着奥妥珠单抗的广泛应用，其特有的安全性问题逐渐凸显。奥妥珠单抗诱导的急性血小板减少（OIAT）在GALLIUM研究中国亚组中发生率高达40.0％[8]。OIAT多发生于首次给药后48 h内，起病急、恢复相对迅速，不仅可导致治疗延迟或中断，影响治疗强度，严重时还可能增加患者的出血风险[10]。目前OIAT的发生机制尚未完全阐明，且缺乏针对中国人群的危险因素研究[10]–[11]，关于其危险因素及管理策略的系统性研究仍较为有限[6],[12]。本研究回顾性分析167例一线应用奥妥珠单抗联合化疗的初诊FL患者的临床资料，系统评估OIAT的发生率与动态变化特征，分析其与基线肿瘤负荷及免疫状态的相关性，探讨影响OIAT严重程度的危险因素，以期为增加奥妥珠单抗治疗的安全性提供循证依据。

## 病例与方法

1. 资料收集：本研究是一项单中心、回顾性队列研究。连续纳入2021年1月至2024年12月期间于江苏省人民医院就诊且经世界卫生组织（WHO）分类标准确诊的初诊FL患者。纳入标准：年龄≥ 18岁、既往未接受过抗淋巴瘤治疗、计划并完成一线G-CHOP方案（奥妥珠单抗+环磷酰胺+多柔比星+长春新碱+泼尼松）或GB方案（奥妥珠单抗+苯达莫司汀）治疗，且具有完整的临床及实验室记录。最终共纳入167例患者。本研究经江苏省人民医院伦理委员会审查批准（批件号：2026-SR-513），所有患者均豁免知情同意。研究严格遵循《赫尔辛基宣言》原则。收集患者治疗前基线资料，包括人口学特征、实验室检查（血常规、生化、免疫球蛋白、淋巴细胞亚群、β_2_微球蛋白、IL-10等）、影像学（PET-CT、增强CT）及病理学指标、Ki-67指数、结外受累情况、骨髓浸润情况等。

2. 治疗方案：奥妥珠单抗100 mg（第1个周期第1天）、900 mg（第1个周期第2天）、1 000 mg（第1个周期第8、15天，第2～6个周期第1天），联合化疗方案为G-CHOP方案（21 d为1个周期，共6个周期）或GB方案（28 d为1个周期，共6个周期）；患者后续接受维持治疗：奥妥珠单抗为1 000 mg，每8周1次，维持2年。奥妥珠单抗输注前均给予标准预处理（包括地塞米松、对乙酰氨基酚及抗组胺药物），输注速率遵循药品说明书推荐。

3. 研究终点与疗效评估：研究的主要终点为OIAT的发生率。次要终点包括OIAT的严重程度［依据常见不良反应事件评价标准（CTCAE）5.0分级］、发生时间（从首次用药至PLT开始下降）、PLT恢复时间（从PLT最低值恢复至≥100×10^9^/L或基线75％以上）以及OIAT与基线临床特征的相关性。OIAT定义为首次输注奥妥珠单抗后7 d内发生的PLT下降。在首次奥妥珠单抗用药前及用药后第1、2、3、7天常规监测PLT，在第3个周期及第6个周期治疗结束时采用PET-CT或增强CT进行中期及治疗结束时疗效评估，疗效评估依据Lugano 2014标准。根据FL患者在第1个周期治疗有无发生OIAT分为OIAT与无OIAT两组。根据CTCAE标准5.0对OIAT患者的血小板减少情况进行分级，分为1～2级血小板减少组与3～4级血小板减少组。1～2级血小板减少且未合并出血的OIAT患者根据是否接受治疗分为治疗组与未治疗组。

4. CD20表达情况判断标准：采用流式细胞术检测CD20表达。以CD19/SSC设门圈定B淋巴细胞，以正常B细胞或同型对照为参照，根据CD20的平均荧光强度（MFI）将表达强度分为3级：强阳性定义为MFI≥80％正常B细胞；阳性定义为MFI处于正常B细胞的50％~80％；弱阳性定义为MFI<50％正常B细胞但高于同型对照。

5. 统计学处理：连续变量经Kolmogorov-Smirnov检验评估正态性。符合正态分布的数据以*x*±*s*表示，组间比较采用独立样本*t*检验。非正态分布数据以*M*（*Q*_1_，*Q*_3_）表示，组间比较采用Mann-Whitney *U*检验。分类变量以频数（百分比）表示，组间比较采用卡方检验或Fisher精确检验。采用二元Logistic回归分析发生OIAT的独立危险因素。正态性检验、组间比较以及Logistic回归分析使用SPSS 26.0软件进行，以*P*<0.05为差异具有统计学意义。使用R（版本4.4.1）软件统计数据。

## 结果

1. 临床资料：如表1所示，本研究共纳入167例初诊FL患者，其中男性70例（41.9％），女性97例（58.1％）。中位年龄为53（45，60）岁，Ann Arbor分期Ⅲ～Ⅳ期患者157例（94.0％）。伴B症状52例（31.1％），结外受累77例（46.1％），脾肿大45例（26.9％），病灶长径>7 cm 33例（19.8％），Ki-67指数≥40％ 35例（20.9％）。一线接受G-CHOP方案治疗96例（57.5％），GB方案71例（42.5％）；接受两种方案治疗患者的性别、年龄、FL国际预后指数-2（FLIPI-2）评分、OIAT和非血液学不良事件发生情况等差异均无统计学意义（表1，*P*值均>0.05）。GB方案组患者中性粒细胞百分比、尿酸、D-二聚体及LDH水平均低于G-CHOP方案组，而淋巴细胞百分比及脾肿大患者比例高于G-CHOP方案组（*P*值均<0.05）。此外，G-CHOP方案组中病灶长径>7 cm患者比例更高（26.0％对11.3％，*P*＝0.013）。

**表1:** 167例新诊断滤泡性淋巴瘤患者的基线特征

特征	总体（167例）	G-CHOP方案组（96例）	GB方案组（71例）	统计量	*P*值
男性［例（％）］	70（41.9）	35（36.5）	35（49.3）	2.760	0.096
年龄［岁，*M*（*Q*_1_，*Q*_3_）］	53（45，60）	53（46，62）	54（44，59）	−0.480	0.843
WBC［×10⁹/L，*M*（*Q*_1_，*Q*_3_）］	6.6（5.0，9.1）	6.6（4.7，9.3）	5.6（4.5，8.6）	−1.326	0.185
中性粒细胞百分比［％，*M*（*Q*_1_，*Q*_3_）］	4.1（2.8，6.2）	4.5（3.2，6.7）	3.3（2.6，4.9）	−3.247	0.001
淋巴细胞百分比［％，*M*（*Q*_1_，*Q*_3_）］	1.2（0.8，1.7）	1.1（0.7，1.5）	1.4（0.9，2.0）	−2.475	0.013
HGB（g/L，*x*±*s*）	127.3 ± 19.1	126.8 ± 19.8	128.1 ± 18.1	−0.467	0.646
PLT（×10⁹/L，*x*±*s*）	181.2 ± 59.1	188.2 ± 60.1	171.8 ± 56.7	1.798	0.077
尿酸［mmol/L，*M*（*Q*_1_，*Q*_3_）］	72.6（58.6，191.5）	80.7（59.6，232.0）	66.3（56.7，85.2）	2.578	0.010
D-二聚体［mg/L，*M*（*Q*_1_，*Q*_3_）］	0.4（0.2，1.0）	0.6（0.2，1.2）	0.3（0.1，0.7）	−1.330	0.003
LDH［U/L，*M*（*Q*_1_，*Q*_3_）］	191.0（162，232.5）	204.5（169.8，247.0）	180.0（159.3，205.8）	−3.284	0.001
铁蛋白［µg/L，*M*（*Q*_1_，*Q*_3_）］	101.5（58.6，207.5）	100.7（57.5，245.5）	104.2（55.4，187.5）	−0.616	0.538
β_2_微球蛋白（mg/L，*x*±*s*）	2.6 ± 1.2	2.5 ± 1.1	2.8 ± 1.3	−1.262	0.207
IL-10［ng/L，*M*（*Q*_1_，*Q*_3_）］	5.4（2.9，11.9）	5.53（3.1，28.2）	5.2（2.8，10.4）	−0.122	0.385
IgM（g/L，*x*±*s*）	1.2 ± 3.5	1.5 ± 4.6	0.8 ± 1.0	−2.387	0.017
IgA［g/L，*M*（*Q*_1_，*Q*_3_）］	1.4（1.0，2.1）	1.5（1.1，2.1）	1.3（0.9，2.1）	−1.559	0.119
CD34^+^T细胞［个/µl，*M*（*Q*_1_，*Q*_3_）］	71.7（60.3，80.0）	71.2（63.3，77.5）	72.7（57.1，84.4）	−0.703	0.410
CD4^+^T细胞（个/µl，*x*±*s*）	35.1 ± 14.1	35.9 ± 12.0	34.1 ± 16.3	0.736	0.451
CD8^+^T细胞［个/µl，*M*（*Q*_1_，*Q*_3_）］	23.5（16.7，32.2）	24.3（17.2，32.8）	21.8（14.4，31.5）	−0.038	0.960
Ann Arbor分期［例（％）］					0.409
Ⅰ～Ⅱ期	10（6.0）	7（7.3）	3（4.2）	0.250	
Ⅲ～Ⅳ期	157（94.0）	89（92.7）	68（95.8）		
FLIPI-2评分分层［例（％）］				0.003	0.880
低风险（0～1分）	66（39.5）	38（39.6）	28（39.4）		
中风险（2分）	59（35.3）	34（35.4）	25（35.2）		
高风险（3～5分）	42（25.2）	24（25.0）	18（25.4）		
B症状［例（％）］	52（31.1）	29（30.2）	23（32.4）	0.091	0.763
结外受累［例（％）］	77（46.1）	46（47.9）	31（43.7）	0.295	0.586
HGB≤120 g/L［例（％）］	62（37.1）	39（40.6）	23（32.4）	1.182	0.276
β_2_微球蛋白>3 mg/L［例（％）］	42（25.1）	21（21.9）	21（29.6）	1.290	0.257
骨髓受累［例（％）］	95（56.9）	50（52.1）	45（63.4）	2.120	0.097
受累淋巴结区域≥3个［例（％）］	143（85.6）	79（82.3）	64（90.1）	2.040	0.113
脾肿大［例（％）］	45（26.9）	20（20.8）	25（35.2）	4.293	0.032
病灶长径>7 cm［例（％）］	33（19.8）	25（26.0）	8（11.3）	5.621	0.013
CD20表达情况［例（％）］				1.185	0.015
弱阳性	118（70.7）	74（77.1）	44（62.0）		
阳性	17（10.2）	10（10.4）	7（9.9）		
强阳性	32（19.1）	12（12.5）	20（28.1）		
Ki-67指数≥40％［例（％）］	35（20.9）	25（26.0）	10（14.1）	3.520	0.056
OIAT［例（％）］	65（38.9）	35（36.5）	30（42.3）	0.580	0.263
非血液学不良事件［例（％）］	37（22.2）	20（20.8）	17（23.9）	0.230	0.368

**注** G-CHOP：奥妥珠单抗+环磷酰胺+多柔比星+长春新碱+泼尼松；GB：奥妥珠单抗+苯达莫司汀；WBC：白细胞计数；HGB：血红蛋白；PLT：血小板计数；LDH：乳酸脱氢酶；IL：白细胞介素；Ig：免疫球蛋白；FLIPI-2：滤泡性淋巴瘤国际预后指数-2；OIAT：奥妥珠单抗诱导的急性血小板减少。符合正态分布的计量资料以*x*±*s*表示，组间比较采用独立样本*t*检验；非正态分布计量资料以*M*（*Q*_1_，*Q*_3_）表示，组间比较采用Mann-Whitney *U*检验（统计量为*Z*值）；计数资料以例（％）表示，组间比较采用*χ*²检验

2. 是否发生OIAT的FL患者基线特征比较：应用奥妥珠单抗治疗后2～3 d连续监测PLT，发生OIAT（OIAT组）65例（38.9％），发生OIAT中位时间为1.0（1.0，2.0）d，PLT下降至最低水平的中位时间为2.0（1.0，3.5）d，PLT中位恢复时间为8.0（6.0，16.0）d。如表2所示，与无OIAT组相比，OIAT组的基线β_2_微球蛋白及IL-10水平更高，FLIPI-2评分为高风险、Ki-67指数≥40％、结外受累及骨髓受累的患者比例更高；中性粒细胞百分比、D-二聚体水平、CD4^+^T细胞数值及IgA水平更低（*P*值均<0.05）。此外，两组的年龄、性别、WBC、HGB、尿酸、血清肌酐、凝血功能、铁蛋白、LDH、补体C3、补体C4、IL-6、肿瘤坏死因子、调节性T细胞、骨髓内巨核细胞计数等差异均无统计学意义（*P*值均>0.05）。

**表2:** OIAT组与无OIAT组滤泡性淋巴瘤患者基线特征比较

特征	OIAT组（65例）	无OIAT组（102例）	统计量	*P*值
男性［例（％）］	28（43.1）	42（41.2）	0.059	0.443
年龄［岁，*M*（*Q*_1_，*Q*_3_）］	52（44，59）	53（44，60）	−0.270	0.135
WBC［×10⁹/L，*M*（*Q*_1_，*Q*_3_）］	5.5（4.5，8.8）	6.6（5.0，9.1）	−1.640	0.101
中性粒细胞百分比［％，*M*（*Q*_1_，*Q*_3_）］	3.4（2.5，4.9）	4.3（3.1，6.6）	−2.780	0.005
淋巴细胞百分比［％，*M*（*Q*_1_，*Q*_3_）］	1.2（0.9，2.0）	1.2（0.8，1.7）	−1.129	0.259
HGB（g/L，*x*±*s*）	126.4 ± 22.5	129.1 ± 18.6	0.828	0.389
PLT（×10⁹/L，*x*±*s*）	144.1 ± 55.4	137.7 ± 47.9	6.231	<0.001
网织红细胞百分比（％，*x*±*s*）	3.3 ± 8.5	1.7 ± 0.6	1.223	0.029
尿酸［mmol/L，*M*（*Q*_1_，*Q*_3_）］	72.4（54.6，112.2）	69.6（59.0，159.8）	−0.202	0.840
D-二聚体［mg/L，*M*（*Q*_1_，*Q*_3_）］	0.2（0.1，0.7）	0.6（0.3，1.3）	−2.984	0.001
LDH［U/L，*M*（*Q*_1_，*Q*_3_）］	188.0（154.0，232.0）	191.0（160.0，229.5）	−0.447	0.655
铁蛋白［µg/L，*M*（*Q*_1_，*Q*_3_）］	100.7（55.8，167.2）	114.0（60.6，252.9）	−0.954	0.340
β_2_微球蛋白（mg/L，*x*±*s*）	2.7（1.8，3.9）	2.2（1.7，3.0）	−2.260	0.024
IL-10［ng/L，*M*（*Q*_1_，*Q*_3_）］	8.0（3.7，32.0）	4.6（2.4，7.9）	−2.642	0.008
IgM［g/L，*M*（*Q*_1_，*Q*_3_）］	0.6（0.5，1.0）	0.8（0.6，1.1）	−1.874	0.061
IgA［g/L，*M*（*Q*_1_，*Q*_3_）］	1.2（1.0，2.1）	1.6（1.2，2.2）	−1.959	0.045
CD34^+^T细胞［个/µl，*M*（*Q*_1_，*Q*_3_）］	71.3（38.9，78.2）	71.4（62.6，80.6）	−1.415	0.157
CD4^+^T细胞（个/µl，*x*±*s*）	32.4 ± 17.6	37.4 ± 11.8	1.845	0.043
CD8^+^T细胞（个/µl，*x*±*s*）	21.3 ± 16.6	26.8 ± 12.7	2.128	0.024
FLIPI-2评分分层［例（％）］			35.862	<0.001
低风险	24.0（36.9）	84.0（82.4）		
高风险	41.0（63.1）	18.0（17.6）		
结外受累［例（％）］	35.0（53.8）	42.0（41.2）	2.565	0.045
Ann Arbor分期［例（％）］			0.005	0.616
Ⅰ～Ⅱ期	4.0（6.2）	6.0（5.9）		
Ⅲ～Ⅳ期	61.0（93.8）	96.0（94.1）		
骨髓受累［例（％）］	41（63.5）	54（52.9）	1.659	0.197
脾肿大［例（％）］	24（36.9）	21（20.6）	5.381	0.020
Ki-67指数≥40％［例（％）］	19（29.2）	16（15.7）	4.397	0.036
病灶长径>7 cm［例（％）］	11（16.9）	22（21.6）	0.540	0.462

**注** OIAT：奥妥珠单抗诱导的急性血小板减少；WBC：白细胞计数；HGB：血红蛋白；PLT：血小板计数；LDH：乳酸脱氢酶；IL：白细胞介素；Ig：免疫球蛋白；FLIPI-2：滤泡性淋巴瘤国际预后指数-2。符合正态分布的计量资料以*x*±*s*表示，组间比较采用独立样本*t*检验；非正态分布计量资料以*M*（*Q*_1_，*Q*_3_）表示，组间比较采用Mann-Whitney *U*检验（统计量为*Z*值）；计数资料以例（％）表示，组间比较采用*χ*²检验

对相关临床因素与OIAT发生的相关性进行二元Logistic回归分析，结果显示，结外受累（*OR*＝3.395，95％ *CI*：1.229～9.378，*P*＝0.018）、FLIPI-2评分>3分（*OR*＝3.622，95％ *CI*：1.192～10.973，*P*＝0.023）、基线PLT<150×10^9^/L（*OR*＝3.216，95％ *CI*：1.122～9.215，*P*＝0.030）是发生OIAT的独立危险因素。

3. 不同严重程度OIAT患者的临床特征、转归及复发情况比较：65例OIAT患者中1～2级血小板减少47例（72.3％）、3～4级血小板减少18例（27.7％）。与1～2级血小板减少组相比，3～4级血小板减少组初诊时骨髓受累［77.8％（14/18）对57.4％（27/47），*P*＝0.001］、结外受累［66.7％（12/18）对53.2％（25/47），*P*＝0.030］、脾肿大［50.0％（9/18）对31.9％（15/47），*P*＝0.007］以及发生非血液学不良事件［72.2％（13/18）对40.4％（19/47），*P*<0.001］的患者比例更高。两组PLT下降时间差异无统计学意义［中位数：1.0（1.0，1.5）d对1.0（1.0，2.0）d，*P*＝0.075）］，但发生3～4级血小板减少的患者较1～2级患者的PLT恢复时间更长［中位数：16.0（8.0，18.5）d对7.0（5.0，12.8）d，*P*＝0.010）］（**扫描本文首页二维码查阅附表1**）。3～4级血小板减少患者后续疗程中再次发生OIAT的比例更高（72.2％对44.7％，*P*<0.001），而更换为利妥昔单抗的比例差异无统计学意义（2.1％对0，*P*＝0.249）。

4. 升血小板治疗对OIAT转归的影响：本队列中发生1～2级血小板减少的患者共47例，均未出现活动性出血；17例（36.2％）采取了升血小板治疗（治疗组），其中10例为重组人血小板生成素注射液治疗、4例为海曲泊帕治疗、1例为升血小板胶囊治疗、2例为重组人血小板生成素注射液联合海曲泊帕治疗。1～2级血小板减少的患者中治疗组的PLT恢复时间较未治疗组并未缩短［中位数：7.1（5.0，12.5）d对7.4（5.2，13.0）d，*P*＝0.338］。18例3～4级血小板减少患者：12例3级患者中有2例发生出血事件（2/12，16.7％），6例4级患者中有1例发生出血事件（1/6，16.7％）；3例（16.7％）采取了升血小板治疗，其中2例为海曲泊帕治疗、1例为重组人血小板生成素注射液联合海曲泊帕治疗，所有患者均出现好转。3～4级血小板减少患者中治疗组的PLT平均恢复时间较未治疗组并未缩短［（21.0 ± 14.4）d对（15.5 ± 9.9）d，*P*＝0.420）］。

5. 再次发生OIAT的特征及临床干预：在65例发生OIAT的患者中，共有41例（63.1％）在后续治疗周期中再次发生OIAT。再次发生OIAT主要集中于治疗早期，其中第2个疗程发生29例（44.6％）、第3个疗程25例（38.5％）、第4个疗程21例（32.3％）、第5个疗程22例（33.8％）、第6个疗程23例（35.4％）、第7个疗程及第9个疗程分别为3例（4.6％）和1例（1.5％）。在复发频率方面，复发1次12例（18.5％）、2次5例（7.7％）、3次6例（9.2％）、4次7例（10.8％）、5次10例（15.4％）、6次1例（1.5％），提示部分患者存在多次反复发生的特点。OIAT发生的中位时间为2.0（1.0，2.0）d，发生时中位PLT最低值为76.0（52.0，93.0）×10⁹/L，PLT恢复的中位时间为3.0（2.0，4.0）d。在治疗干预方面，复发后接受药物干预次数为63次（50.8％，63/124）、未干预次数为61次（49.2％，61/124）。

## 讨论

本研究回顾性分析了167例接受奥妥珠单抗联合化疗的初治FL患者发生OIAT的临床特征。结果显示，OIAT发生率为38.9％，其中3～4级血小板减少占27.7％。OIAT呈现急性、一过性特征，中位发生时间为用药后1 d，中位恢复时间为8 d。结外器官受累、FLIPI-2评分>3分及基线PLT<150×10^9^/L是发生OIAT的独立危险因素。根据严重程度分层，1～2级OIAT患者预后良好，而3～4级患者PLT恢复时间更长、发生非血液学不良事件比例更高，需积极干预。本研究观察到的OIAT发生率（38.9％）与GALLIUM中国亚组结果（约40.0％）接近[8],[13]，但高于全球人群（11.4％）[6]，差异与患者特征、化疗方案及监测频率有关[14]–[17]。奥妥珠单抗联合伊布替尼[18]、ViPOR[19]及MIL62[20]等研究均报道OIAT常见。上述研究提示，OIAT为奥妥珠单抗类药物共有、时间分布特征明显的类效应，治疗初期需高度警惕。

本研究多因素分析提示，OIAT的发生可能与高肿瘤负荷及低免疫状态均相关，OIAT组结外受累、FLIPI-2高危、Ki‑67指数≥40％、脾肿大及β_2_微球蛋白升高等反映肿瘤高负荷的指标更高，而基线CD4⁺T细胞计数与IgA水平较低，提示免疫状态相对抑制。本研究结果提示，OIAT的发生并非单一因素作用，而是在肿瘤负荷与免疫背景交互影响下的临床表型。这与接受奥妥珠单抗联合BCL2抑制剂维奈克拉固定周期治疗的慢性淋巴细胞白血病患者常伴有免疫缺陷状态一致[21]。OIAT作为奥妥珠单抗的类效应，其发生可能与患者的基础免疫微环境密切相关，而非完全取决于联合使用的化疗药物。我们观察到OIAT组IL-10水平显著升高，提示细胞因子释放可能在药物诱导的急性血小板破坏中起作用[22]。此外，骨髓受累及脾肿大导致造血微环境被侵占，降低血小板生成储备，使其对药物冲击更为敏感[23]。而基线免疫功能的下降可能削弱对治疗相关炎症反应的调节，进一步加剧血小板消耗。

本研究识别出结外受累、FLIPI-2评分>3分和基线PLT<150×10⁹/L 3个独立危险因素。临床可考虑建立简易风险分层模型，对具备多项危险因素的患者在用药后24～72 h内加强血小板监测，并预先制定干预策略。根据严重程度分层分析发现，1～2级OIAT患者（72.3％）血小板恢复较快（中位恢复时间为7 d），且在本研究中，对无出血的OIAT患者给予升血小板治疗并未缩短PLT恢复时间，支持采取以观察为主的策略。而3～4级OIAT患者PLT恢复时间显著延长（中位恢复时间为16 d），合并非血液学不良事件比例高，需积极干预，包括使用血小板生成素受体激动剂类药物并密切监测。

对后续疗程治疗中再次发生OIAT进行统计，结果显示在肿瘤负荷明显降低的情况下，OIAT在后续疗程中虽多次发生，但程度为1～2级，提示OIAT发生率和严重程度与肿瘤负荷呈现正相关趋势。药物本身引起的免疫稳态崩溃与记忆性自身免疫也是重要原因。第1个疗程后肿瘤显著缓解，当药物持续存在时，T细胞对自身抗原的反应性不会同步消失。因此，OIAT的发生率与自身反应性T细胞克隆的扩增强度相关。

本研究存在局限性：作为一项单中心回顾性研究，存在固有的选择偏倚；尽管总体样本量适中，但3～4级OIAT患者仅18例，限制了该亚组的深入分析与统计检验效能；缺乏长期随访数据，无法评估OIAT对患者远期无进展生存和总生存的潜在影响。此外，对机制的探讨仍基于临床指标的相关性分析，缺乏分子生物学或体外实验层面的直接证据。

综上，OIAT是FL治疗中不可忽视的不良反应，多发生于首次用药后早期，在高肿瘤负荷及免疫状态低下的患者中发生风险显著增高。对于1～2级血小板减少可以观察为主，而3～4级血小板减少需警惕合并非血液学不良事件并积极干预，临床医师需根据基线特征分层管理，对高风险患者加强监测并及时干预。未来研究应聚焦于机制解析和个体化治疗策略的优化，以平衡疗效与安全性。

## Supplementary Material


